# Bacteriology of community acquired pneumonia in adult patients at Felege Hiwot Referral Hospital, Northwest Ethiopia: a cross-sectional study

**DOI:** 10.1186/s13756-019-0560-0

**Published:** 2019-06-14

**Authors:** Dinbere Temesgen, Fetlewok Bereded, Awoke Derbie, Fantahun Biadglegne

**Affiliations:** 1 Amhara Public Health Institute (APHI), Bahir Dar, Ethiopia; 2 Department of Medical Laboratory Sciences, College of Medicine and Health Sciences, Bahir Dar University, P.O.BOX: 1383, Bahir Dar, Ethiopia; 3 Center for Innovative Drug development and Therapeutics Trials for Africa (CDT-Africa), Addis Ababa University, Addis Ababa, Ethiopia

**Keywords:** Community acquired pneumonia, Multi drug resistance, Felege Hiwot Referral Hospital, Northwest Ethiopia

## Abstract

**Background:**

Community acquired pneumonia (CAP) is reported as a common cause of lower respiratory tract infection worldwide. Bacterial pathogens and antimicrobial resistance (AMR) associated with this infection varied between geographical regions. Knowledge of the pathogens in a given area and their up-to-date AMR profile is essential for optimal management of patients. This study was aimed at assessing the type of bacterial isolates and their AMR among CAP adult patients at the Felege Hiwot Referral Hospital (FHRH), Northwest Ethiopia.

**Methods:**

This cross-sectional study was conducted during 1 April to 30 July 2018. Demographic related data were collected from the study participants using a structured questionnaire. Sputum samples were collected and processed to identify pathogens using the conventional culture and biochemical tests as per the standard procedures. The Kirby Bauer disk diffusion method was implemented for the AMR testing. Descriptive and multivariable analysis was conducted using SPSS version 23.

**Results:**

Among 414 presumptively diagnosed study participants for CAP, bacterial pathogens were identified from 167 (40.3%) participants. Among these, multidrug resistance (MDR) accounted for 127(76%) of the isolates. The predominant isolates were *Streptococcus pneumoniae* at 60(35.9%) and *Klebsella pneumoniae* at 30(18%). Overcrowded living conditions [AOR 1.579 (95%CI: 1.015–2.456)] and alcohol use [AOR 4.043 (95% CI, 2.537–6.443)] were found statistically associated with culture positive sputum.

**Conclusions:**

The study showed high prevalence of mono- and multi**-**drug resistant isolates in the study area. Therefore, regular surveillance of the type of isolates and their AMR patterns should be considered. Interventions for reducing community acquired pneumonia should be integrated with lifestyle factors related to household and alcohol use.

## Background

Pneumonia is reported as an acute respiratory tract infection evidenced with a clinical and/or radiological consolidation of the lungs [1]. According to Sligl and Marrie report, pneumonia developed due to the reduction of the defensive mechanisms of the lung. Pneumonia is transmitted through inhalation or aspiration of the pathogens [2]. Pneumonia, which is usually caused by bacteria, is the most common cause of hospital attendance in adults of the developing countries [1]. Van Gageldonk-Lafeber and his colleagues also showed that children and elders are highly affected by the disease [3].

Pneumonia is usually classified as community and hospital acquired or those occurred in patients with underlying damaged lung including suppurative and aspiration pneumonia [1]. According to Prina and his collegues report in 2015, community acquired pneumonia (CAP) is an infection of the lung parenchyma which is not acquired from a health care system [4]. The disease was reported as a major health problem causing morbidity and mortality worldwide [5]. In Africa, the mortality rate among adult patients varied between 6 and 15% [6]. In South Africa, CAP was the fifth-largest killer reported at 3.9% of all deaths [7]. Previous reports showed that the prevalence of bacterial CAP in Ethiopia was varied between 42.9 and 50% [8–10].

Studies showed that old age, smoking, alcoholism, immunosuppressive conditions, chronic obstructed pulmonary diseases (COPD) were reported as key factors for the development of the disease [1, 11, 12]. Furthermore, asthma, cardiovascular disease, chronic liver or renal disease, diabetes mellitus, overcrowded living condition, and recent upper respiratory tract infection (URTI) were reported predictors of the CAP.

In Ethiopia, the diagnosis of CAP is relied on the medical history and physical examinations of the suspected individuals. However, the clinical characteristics of CAP cannot be consistently used to establish the etiologic diagnosis with adequate sensitivity and specificity. So, appropriate bacteriological diagnosis is very important [13, 14]. The bacterial pathogens associated with the CAP and their antimicrobial resistance pattern varied from place to place and in time [15]. Previous studies on this regard reported that *Streptococcus pneumoniae* (*S. pneumoniae)* was the most commonly isolated pathogen followed by *Haemophilus influenzae* (*H. influenzae*), *Staphylococcus aureus* (*S. aureus*), *Chlamydia pneumoniae*, *Legionella* species, and *Mycoplasma pneumonia*e [3, 16]. In Ethiopia, *S. pneumoniae* is the most frequent isolated bacteria followed by *S. aureus* [9, 17]. In these studies, most of the isolated pathogens were found to be resistant to one or more classes of antibiotics.

In developing countries including Ethiopia, treatment of CAP is made usually empirically in which the etiologic agent is rarely identified. So, identifying the most common bacterial pathogens isolated from CAP and their drug resistance profile would be valuable to reduce morbidity and mortality associated with the disease [18]. Therefore, this study was conducted to provide data on the type of bacterial pathogens and their drug resistance profile among CAP adult patients at the Felege Hiwot Referral Hospital (FHRH), Northwest Ethiopia.

## Materials and methods

### Study setting, design and period

This hospital based cross-sectional study was conducted from 1 April to 31 July, 2018 at FHRH, Northwest Ethiopia. The hospital provided specialized services to patients with its different departments. It had more than 400 beds and serving close to 10 million people in the surrounding area. The hospital is located in Bahir Dar, Amhara Regional State, Ethiopia which is about 565 km far from the capital city, Addis Ababa.

### Population, sample size and sampling technique

A total of 414 CAP suspected adult patients were included. A single population proportion method was implemented to determine the sample size [17]. The study subjects were selected using a systematic random sampling technique. Adults, aged ≥18 years that were clinically suspected for CAP and consented to participate in the study were included. However, patients who were under antibiotic treatment and had a history of hospital admission 14 days before the data collection period were excluded from the study.

### Operational definition

In accordance with previous literatures [4, 9, 12, 19, 20] the following terms are defined;

- Community acquired pneumonia (CAP): Pneumonia which is not acquired in a hospital or a long-term care facility.
- Multi-drug resistance (MDR): Resistance to one or more agents in three or more different classes of antimicrobials.
- Overcrowded living condition: Living with more than 10 persons at home.
- Typical symptom of pneumonia: Cough, fever, chills, fatigue, dyspnea, and pleuritic chest pain.

### Data collection and laboratory methods

Data on socio-demographic characteristics and associated factors for culture positive sputum were collected using pretested structured questionnaire.

#### Sample collection and transport

After the patients were instructed how to collect the sample, a total of 414 sputum samples were collected in a disposable, leak proof, sterile and wide mouthed container with tight fitting lid. The collected sputum samples were transported to Amhara Public Health Institute (APHI) microbiology laboratory, which is located close to the hospital, within 20–30 min. Following the standard conventional sputum culture protocol, specimens were first inspected macroscopically and then microscopically for further culture analysis. Those sputum specimens with at least 25 polymorph nuclear leukocytes and less than 10 epithelial cells per low power field were processed for culture. Otherwise, the sputum sample was considered as contaminated with saliva and rejected [17, 21].

### Process of culturing and identification of bacterial isolates

Purulent portion of the sputum specimen was inoculated on MacConkay agar (MAC), Blood agar plate (BAP), and Chocolate agar plate (CAP) using a sterile swab. In order to get pure colonies, the samples were streaked in four quadrants of the plates. The inoculated MAC plates were incubated aerobically at 37 °C for 24 h. However, the inoculated CAP and BAP were incubated using 5% CO_2_ generating candle jar at 37 °C for 24 h. The plates were then examined for growth. Following the standard microbiological procedure, the bacterial isolates were characterized using colony morphology, hemolysis, gram stain, and by means of a panel of biochemical tests. In brief, for gram positive bacteria we used catalase, coagulase, optochin, bile solubility test and for gram negative isolates motility, indole, urea, lysine decarboxylase (LDC), oxidase, triple sugar iron agar (TSI) and citrate utilization tests were performed [9, 22].

### Antimicrobial susceptibility testing (AST)

The Kary-Baur disc diffusion method was used for AST on Muller Hinton agar (MHA) (Oxoid, Ltd., England) as per the Clinical Laboratory Standards Institute (CLSI) guideline. For *S. pneumonia* isolates 5% sheep blood was added on MHA and for those *H. infleunzae* Muller Hinton Chocolate agar was used. Morphologically identical 3 to 5 pure colonies from overnight cultured specimen were suspended in 5 ml sterile nutrient broth (Oxoid, Ltd.,England) and mixed thoroughly to make the suspension homogenous. The inoculum turbidity was adjusted to 0.5 McFarland standards [22]. Then, the bacterial suspensions were seeded on the surface of the MHA using a sterile cotton swab and allowed to dry for about 3 to 15 min. The antimicrobial impregnated disks were placed on the media using sterile forceps in such a way that each disk was placed at least 24 mm away from each other to avoid overlapping of zone of inhibition. After the disk was placed on the inoculated media, the plate was allowed to stand for 15 min, so that the antibiotic diffused into the media. The plates were incubated at 37^o^C for 24 h and the zone of inhibition was measured and interpreted as sensitive, intermediate and resistant as per the CLSI protocol [22, 23].

Based on the CLSI guideline, we used Ceftriaxone (CRO, 30 μg), Ciprofloxacin (CIP, 5 μg), Tetracycline (TE, 30 μg), Chloramphenicol (C, 30 μg), Erythromycin (E, 15 μg), Doxycycline (DO, 30 μg), Penicillin (P, 10 μg), Trimethoprim-sulfamethoxazole (TMP-SMX, 1.25 + 23.75 μg), Oxacillin (OXA, 1 μg) and Clindamycin (DA, 2 μg) for gram positive isolates. Whereas, for gram negatives, we used Gentamycin (CN, 10 μg), Ampicillin (AMP, 10 μg), Amoxacilin-Clavunic acid (20/10 μg), Trimethoprim-sulfamethoxazole (TMP-SMX, 1.25 + 23.75 μg), Ceftriaxone (CRO, 30 μg), Doxycycline (DO, 30 μg), Tetracycline (TE, 30 μg), Ciprofloxacin (CIP, 5 μg), and Chloramphenicol (C, 30 μg). In addition to these, Piperacilin (PIP, 100 μg) and Ceftazidime (CAZ, 30 μg) were used for *P. aeruginosa* [23]. All antibiotics were obtained from Abtek Biologicals Ltd., UK.

### Quality control

We strictly followed the manufacturers’ instruction and bacteriological standard procedures during culture media preparation and AST testing. The standard reference bacteria strains such as *S. aureus* (ATCC®25,923), *H. influenzae* (ATCC® 49,247) and *S. pneumoniae* (ATCC® 49,619) were used as a quality control. Moreover, the whole procedure and result interpretation was cross-checked by a senior medical microbiologists working at APHI.

### Data organization, processing and analysis

All data were entered, cleared, and analyzed using the SPSS statistical software package, Version 23.0 *(IBM Corp. Released 2011. IBM SPSS Statistics for Windows. Armonk, NY: IBM Corp.).* Descriptive data analysis was used to visualize differences with in the data. Frequencies, odd ratio (OR) with its 95% confidence interval (CI) were calculated. All covariates that were associated with the outcome variable in the bivariate analysis were subsequently included in the multivariable analysis to determine factors associated with culture positive sputum. A *P* value less than or equal to 0.05 was considered to show statistically significant differences.

## Results

### Demographic characteristics

A total of 414 CAP suspected adult patients were enrolled in the study. Among these, 239 (57.7%) were males and 191(46.1%) were in the age group of 36–49 years with mean age of the participants at 42.4 years (standard deviation ±13.8). Moreover, 10(2.4%) of the participants had asthma and 12(2.9%) of them were smokers. The demographic characteristic of the study participants is summarized in Table 1.

**Table 1:** Socio-demographic characteristics of the study participants at FHRH, 2018

Variables		Frequency (%)
Sex	Male	239 (57.7)
	Female	175 (42.3)
Age in years	18–35	91 (22)
	36–49	191 (46.1)
	50–64	98 (23.7)
	≥ 65	34 (8.2)
Residence	Rural	256 (61.8)
	Urban	158 (38.2)
Occupation	Government Employee	65 (15.7)
	Farmer	208 (50.2)
	Merchant	46 (11.1)
	House wife	50 (12.1)
	Daily laborer	21 (5.1)
	Student	24 (5.8)
Educational Status	No education	249 (60.1)
	1–8 Grade	82 (19.8)
	9–12 Grade	60 (14.5)
	Diploma and above	23 (5.6)
Alcohol use	Yes	168 (40.6)
	No	246 (59.4)
Smoking	Yes	12 (2.9)
	No	402 (97.1)
Asthmatic case	Yes	10 (2.4)
	No	404 (97.60
Crowded living condition	Yes	198 (47.8)
	No	216 (52.2)

### Distribution of the bacterial growth and types of the isolates

**Fig. 1:**
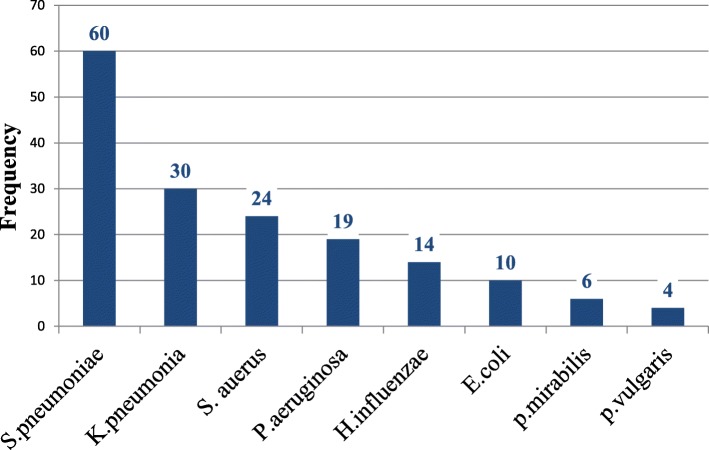
The distribution of bacterial isolates identified from the participants at FHRH, 2018

The overall prevalence of culture positive sputum was at 167 (40.3, 95% CI; 35.5–44.9). The distribution of the isolates is summarized in Fig. 1. The most frequently identified bacteria was *S. pneumoniae* at 60 (35.9%) followed by *K. pneumoniae* at 30 (18%), *S. aureus* at 24 (14.4%), *P. aeruginosa* at 19 (11.4%) and *H. influenzae* at 14(8.4%). In this study, gram positive and gram negative isolates constituted 84 (50.3%) and 83 (49.7%), respectively.

### Antimicrobial resistance profile of the isolates

The overall AMR profile of the isolates is presented in Table 2. In the present study *S. pneumoniae* showed higher level of resistance to Oxacillin at (56.7%) and Tetracyclin at (45%). However, (96.7%) of the *S. pneumoniae* were found sensitive to Chloroamphenicol. Similarly (96.7%) of the *S. pneumoniae* isolates were found sensitive to Erythromycin and all of the *S. pneumoniae* were found sensitive to Ceftriaxone. Moreover, our study showed that (96.7%) of the *K. pneumoniae* isolates were resistant to Amoxacilin-Clavunic acid. Further, (90%) of the *K. pneumoniae* isolates were found resistant to Trimethoprim-Sulfamethoxazole and all of the *K. pneumoniae* isolates were resistant to Tetracycline and Ampicillin. However, (96.7%) and (100%) of these isolates were found sensitive to Ciprofloxacilin and Ceftriaxone, respectively.

**Table 2:** Antimicrobial resistance profile of the isolated organisms at FHRH, 2018

Antimicrobialtested	Bacterial isolates (N)							
	*S.pneumoniae* (60)	*K.pneumoniae* (30)	*S. auerus*(24)	*P.aeruginosa* (19)	*H.influenzae* (14)	*E.coli* (10)	*P.mirabilis* (6)	*P.*vulgaris(4)
N (%)E	2 (3.3)	NA	11 (45.8)	NA	NA	NA	NA	NA
P	NA	NA	18 (75)	NA	NA	NA	NA	NA
OXA	34 (56.7)	NA	18 (75)	NA	NA	NA	NA	NA
TMP-STX	5 (8.3)	27 (90)	21 (87.5)	NA	13 (92.9)	8 (80)	5 (83.3)	4 (100)
CRO	0 (0)	0 (0)	NA	5 (26.3)	4 (28.6)	0 (0)	3 (50)	1 (25)
DA	5 (8.3)	NA	2(8.3)	NA	NA	NA	NA	NA
DO	23(38.3)	6(20)	10(41.7)	NA	NA	9 (90)	5 (83.3)	4(100)
TE	27 (45)	30(100)	22(91.7)	NA	14(100)	9(90)	6(100)	4(100)
AMP	NA	30 (100)	NA	NA	6 (42.9)	7(70)	5(83.3)	4(100)
CN	NA	2 (6.7)	NA	11(57.9)	NA	2(20)	5 (83.3)	4(100)
AMC	NA	29(96.7)	NA	NA	0 (0)	8(80)	0 (0)	(0)
PIP	NA	NA	NA	3(15.8)	NA	NA	NA	NA
CIP	NA	1 (3.3)	7 (29.2)	4 (21.1)	5 (35.7)	0 (0)	4 (66.7)	1(25)
CAZ	NA	NA	NA	1 (5.3)	NA	NA	NA	NA
C	2 (3.3)	6 (20)	7 (29.2)	NA	5 (35.7)	9 (90)	5 (83.3)	4(100)

Note: *NA* Not applicable, *CRO* Ceftriaxone, *CIP* Ciprofloxacilin, *TE* Tetracycline, *C* Chloramphenicol, *E* Erythromycin, *DO* Doxycycline, *P* Penicillin, *CN* Gentamycin, *TMP- STX* Trimethoprim-sulfamethoxazole, *AMP* Ampicillin, *OXA* Oxacillin, *CAZ* Ceftazidime, *AMC* Amoxacilinclavunic acid, *DA* Clidamycin, *PIP* Piperacilin

Interestingly, in this study 127 (76%) of the isolates showed resistance to three and more classes of antimicrobials; (Multi-drug Resistant). Specifically, 33 (55%) of *S. pneumoniae*, 30(100%) of *K.pneumoniae,* 24(100%) of *S. auerus,* 8 (42.1%) of *P. aeruginosa*, 14 (100%) of *H. influenzae,* 9 (90%) of *E.coli,* 5 (83.3%) of *P. mirabilis* and 4 (100%) of *P. vulgaris* isolates were found MDR (Table 3).

**Table 3:** Multi-drug resistance (MDR) profile of the isolates at FHRH, 2018

Bacteria isolated	Degree of resistance									Total MDR isolates ≥ R3
	R0 (%)	R1 (%)	R2 (%)	R3 (%)	R4 (%)	R5 (%)	R6 (%)	R7 (%)	R8 (%)	
*S.pneumoniae* (*n* = 60)	2(3.3)	25(41.7)	_	8(13.3)	22(36.7)	_	3 (5)	_	_	33(55)
*K.pneumoniae* (*n* = 30)	_	_	_	_	1(3.3)	22(73.3)	7(23.3)	_	_	30(100)
*S.aureus* (*n* = 24)	_	_	_	2(8.3)	10(41.7)	7(29.2)	5(20.8)	_	_	24(100)
*P.aeruginosa* (*n* = 19)	_	6(31.6)	5(26.3)	5(26.3)	2(10.5)	1(5.3)	_	_	_	8(42.1)
*H.infleuenzae* (*n* = 14)	_	_	_	8(57.1)	_	1(7.1)	2(14.3)	3(21.4)	_	14(100)
*E.coli* (*n* = 10)	_	1(10)	_	1(10)	1(10)	_	5(50)	2(20)	_	9(90)
*P.mirabilis* (*n* = 6)	_	_	1(16.7)	_	_	_	_	2(33.3)	3(50)	5(83.5)
*P.vulgaris* (*n* = 4)	_	_	_	_	_	_	_	3(75)	1(25)	4(100)
Total (*n* = 167)	2(1.2)	32(19.2)	6(3.5)	24(14.4)	36(21.5)	31(18.6)	22(13.2)	10(6)	4(2.4)	127(76)

Note: R0: susceptible to all antibiotics, R1–R8: resistance to 1, 2, 3, 4, 5, 6, 7 & 8 antibiotics, ≥ R3: resistance to 3 or more antibiotic, MDR: multidrug resistance

### Factors associated with culture positive sputum

The multivariable analysis showed that overcrowded living condition [AOR 1.579 (95%CI: 1.015–2.456)] and alcohol use [AOR 4.043 (95% CI: 2.537–6.443)] were significantly associated with sputum culture positive result (Table 4).

**Table 4:** Factors associated with culture positive sputum at FHRH, 2018

Variables		Culture					
		Positive	Negative	COR(95% CI)	*p*-value	AOR(95% CI)	*p*-value
Sex	Male	100	139	1.160(0.778,1.728)	0.466		
	Female	67	108				
Age	18–35	35	54				
	36–49	85	108	1.199(0.722,1.994)	0.483		
	50–64	32	66	0.741(0.408,1.344)	0.323		
	≥ 65	15	19	1.206(0.544,2.676)	0.645		
Occupation	Government employee	31	34				
	Farmer	67	141	0.521(0.296,0.919)	0.024	0.390(0.105,1.450)	0.160
	Merchant	25	21	1.306(0.612,2.784)	.490		
	House wife	22	28	0.862(0.411,1.808)	0.694		
	Daily laborer	15	6	2.742(0.946,7.950)	0.063	1.796(0.481,6.709)	0.384
	Student	7	17	0.452(0.165,1.235)	0.121	0.606(0.165,2.229)	0.451
Educational status	No education	88	161	1.025(0.418,2.512)	0.957		
	1–8 grade	40	42	1.786(0.683,4.669)	0.237		
	9–12 grade	31	29	2.004(0.740,5.428)	0.171	2.228(0.728,6.820)	0.160
	Diploma and above	8	15				
Residence	Rural	85	171	0.461(0.307,0.692)	0.000	0.638(0.283,1.439)	0.279
	Urban	82	76				
Over Crowded living condition	Yes	94	104	1.771(1.191,2.632)	0.005	1.579 (1.015,2.456)	0.043
	No	73	143				
Alcohol use	Yes	97	71	3.435(2.273,5.190)	0.000	4.043(2.537,6.443)	0.000
	No	70	176				

## Discussion

In our study the overall prevalence of culture positive sputum among adults was at 40.3%. Studies conducted in Ethiopia and elsewhere in Africa and Europe have reported the prevalence of culture positive sputum among CAP suspected patients varied between 42 and 47.2% [9, 10, 15, 24, 25]. The design of the studies including sample size and study subjects as well as the geographical variances might contribute for the difference of the results.

In the present study the predominant isolate was *S. pneumoniae* at 60 (35.8%) followed by *K. pneumonia* at 30 (18%) and *S. aureus* at 24 (14.4%). This result is inconsistent with other studies conducted in Ethiopia and others with different geographical regions of the world [1, 10, 25, 26]. A study in Jimma, Ethiopia for example reported lower proportion of *S. pneumoniae* isolates at (28.3%) [9]. Similarly, our finding was significantly higher compared to a study reported in India at (3.4%) [26], Iran at (25.8%) [24], Estonia at (28.1%). However, other studies done in China and other countries reported higher proportion of *S. pneumoniae* ranging from 36.4 to 66% [1, 27, 28]. Likewise, in the present study, *K. pneumoniae and S. aureus* were the second and the third most frequently identified isolates that accounted 18 and 14.4%, respectively. Comparable findings have been reported by previous studies done in Ethiopia and abroad like Nigeria, China and India [1, 9, 10, 15, 24–26, 28].

In this study, we noted that 84 (50.3%) of the isolates were gram positives while 83 (49.7%) constitute the gram negatives. A study conducted in Ethiopia reported gram positive bacteria at 52.1% and gram negative isolates at 47.9% [10] from similar study participants. Equally, a study in Ghana reported comparable proportion at 40% gram positive and 58% gram negatives isolates [29]. The varying proportion of isolates among these studies might be attributed due to the variation in the geographical distribution of the isolates, sample size and the specific methodological issues.

In this study, *S. pneumoniae* was the most common isolate at 56.7% found to be resistance to Oxacillin. However, 96.7% of these isolates were found susceptible to Erythromycin. Previous studies conducted in Ethiopia and Europe had reported similar findings on this regard [9, 10, 22]. In this study we also noticed that *K. pneumoniae* showed 100% resistance to Tetracycline and 96.7% to Amoxacilin-Clavunic acid. This is in line with the studies conducted in Arbamich, Ethiopia [10], Nigeria [25] and Bangladesh [1]. In addition, in the present study we noted high level of antimicrobial resistance to Tetracycline, Ampicillin, and Trimethoprim-sulfamethoxazole. This might be related with a high rate of prescription and self-medication of these drugs in the study area. On the other hand, most of the isolates in this study were susceptible for Ceftriaxone.

The overall prevalence of MDR was at 76% which is slightly higher as compared to the studies conducted in Jimma and Arbaminch, Ethiopia where the prevalence was at 62.7% [9] and 60.3% [10], respectively. MDR in resource constraint settings is highly contributed by the widespread misuse of antimicrobials by patients due to lack of access to appropriate treatment and under use of drugs due to inadequate dosing or incomplete treatment courses. The other factor contributed to MDR might be over use of drugs while the infectious pathogen is not well characterized due to the absence of well-organized bacteriology laboratory in the study area. In Ethiopia, it is very common to buy and use antimicrobials and other drugs from private pharmacies without prescription. These all could play a role in the increasing trend of antimicrobial resistance in the different health settings of Ethiopia in general and our study site in particular.

Our study measured factors associated to culture positive sputum among CAP suspected patients. The study results showed that overcrowded living condition [AOR 1.579 (95%CI: 1.015–2.456)] and alcohol use [AOR 4.043 (95% CI: 2.537–6.443)] were found significantly associated. Similar couple of studies done in Europe reported that risk for culture positive sputum was increased among individuals who have been consuming alcohol and living in a household of over 10 people [12, 30] the latter may results in indoor air pollution. A recently published review paper also reported that alcohol consumption increased the risk of CAP [31]. There are numerous possible instruments to explain the observation that alcohol consumption increased the risk of CAP, including but not limited to the sedative properties of alcohol which can reduce oropharyngeal tone, leading to an increased risk of aspiration of pathogens from the upper respiratory tract. High levels of alcohol intake can also modify the alveolar macrophage function, hence diminishing pulmonary defense against infection [31]. A review by Almirall and his colleagues in 2017 indicated that age, smoking, environmental exposures, malnutrition, previous CAP, chronic bronchitis/chronic obstructive pulmonary disease, asthma, functional impairment, poor dental health, immunosuppressive therapy, oral steroids, and treatment with gastric acid-suppressive drugs were predictors of CAP [32].

This study has provided valuable data on the bacterial isolates and their drug resistance profile among CAP adult patients. Despite this strength, our study has some drawbacks as well; we did not attempt to isolate and identify some common pathogens like *Chlamydia, Mycoplasma, Legionella* species and serotyping was not also done to *H. influenzae* due to resource limitation. In addition, the molecular investigations for the isolated bacteria were not performed. The study also didn’t provide data on serotyping of *S. pneumonaie*, characterizing of methicillin resistant *S. aureus* and extended-spectrum beta-lactamaze as well as carbapenemase-producing organisms detection and analysis.

## Conclusions

In the studied area, relatively higher proportions of *S. pneumoniae, K.pneumoniae* and *S.aureus* were identified. Most of the isolates were found susceptible to Ceftriaxone. However, antimicrobial resistance including MDR was observed to a number of commonly used antibiotics, such as Trimethoprim-sulfamethoxazole, Ampicilin and Tetracyclin. Therefore, as there is no standardized surveillance at FHRH, periodic investigation of the etiologic agents and their antibiotic resistance profile should be made in order to guide clinicians in the management of CAP. Overcrowded living style and alcohol use were found predictors of culture positive sputum. So, interventions for reducing CAP should integrate lifestyle factors related to household and alcohol use. Additional, large scale studies should be considered to further characterize the pathogens involved in CAP.

## Data Availability

Not applicable.

## Abbreviations

APHI: Amhara public health institute
AST: Antimicrobial susceptibility testing
ATCC: American type culture collection
BAP: Blood agar plate
CAP: Community acquired pneumonia
CLSI: Clinical laboratory standard institute
FHRH: Felege hiwot referral hospital
LDC: Lysine decarboxylase agar
LIA: Lysine iron agar
MAC: MacConkey agar
MDR: Multi drug resistant
MHA: Muller hinton agar
MSA: Manitol salt agar
QC: Quality control
SIM: Simon indole motility
SPSS: Statistical package for social sciences
TSI: Triple sugar iron agar
